# Evolution of risk prediction models for post-operative mortality in patients with cirrhosis

**DOI:** 10.1007/s12072-023-10494-0

**Published:** 2023-03-27

**Authors:** Eric Kalo, Jacob George, Scott Read, Avik Majumdar, Golo Ahlenstiel

**Affiliations:** 1 Blacktown Clinical School, School of Medicine, Western Sydney University, Blacktown, NSW 2148 Australia; 2 Blacktown Hospital, Western Sydney Local Health District, Blacktown, NSW 2148 Australia; 3 Storr Liver Centre, The Westmead Institute for Medical Research, Westmead Hospital and University of Sydney, Westmead, NSW 2145 Australia; 4 Victorian Liver Transplant Unit, Austin Health, Heidelberg, VIC 3181 Australia; 5 The University of Melbourne, Melbourne, VIC 3010 Australia

**Keywords:** Surgical risk, Prediction, Cirrhosis, Models, Post-operative, Mortality

## Abstract

The perception of high surgical risk among patients with cirrhosis has resulted in a long-standing reluctance to operate. Risk stratification tools, first implemented over 60 years ago, have attempted to assess mortality risk among cirrhotic patients and ensure the best possible outcomes for this difficult to treat cohort. Existing postoperative risk prediction tools including the Child–Turcotte–Pugh (CTP) and Model for End-stage Liver Disease (MELD) provide some prediction of risk in counselling patients and their families but tend to overestimate surgical risk. More personalised prediction algorithms such as the Mayo Risk Score and VOCAL-Penn score that incorporate surgery-specific risks have demonstrated a significant improvement in prognostication and can ultimately aid multidisciplinary team determination of potential risks. The development of future risk scores will need to incorporate, first and foremost, predictive efficacy, but perhaps just as important is the feasibility and usability by front-line healthcare professionals to ensure timely and efficient prediction of risk for cirrhotic patients.

Metabolic-associated fatty liver disease (MAFLD), alcohol-related liver disease (ARLD), and an aging viral hepatitis population have led to a rise in the prevalence of advanced chronic liver disease. Over time, many will require surgery for a variety of indications, and for whom success is greatly affected by the status of their liver disease. The presence of cirrhosis is associated with increased surgical risk and anesthesia-related complications relative to the general population due to sequalae of portal hypertension and/or liver insufficiency which include: increased risk of infection, predilection to hemostatic dysfunction (bleeding or thrombosis), impaired clearance of numerous drugs, hepatorenal syndrome, splanchnic venous congestion, and systemic vasodilation [1].

Historically, perceptions of high surgical risk for cirrhotic patients have resulted in avoidance of elective surgery, even when the risk was deemed acceptable. The practice of risk aversion is compounded when elective procedures are deferred until a surgical indication becomes emergent, resulting in higher mortality. Existing prediction tools tend to overestimate surgical risk even for surgeries that are safe to perform. Moreover, many mathematical models for risk prediction do not capture factors such as nutritional status, frailty, type, invasiveness of surgical procedure, and presence of concomitant disorders (such as cardiovascular disease, etc.). Consequently, better precision is required to predict post-operative morbidity and mortality for cirrhotic patients.

Tools for risk stratification were first developed to assess mortality risk following portosystemic shunt surgery in patients with cirrhosis. Wantz and Payne first identified three risk classes, A, B, and C [2] that subsequently evolved into the Child–Turcotte–Pugh (CTP) score and the Child class [3, 4]. When applied to other types of intra-abdominal surgeries, patients of CTP Class A had low surgical risk, CTP class B—intermediate risk, while patients with CTP class C were considered as high risk. The CTP class was later superseded in 2000 by the Model for End-Stage Liver Disease (MELD) score. Studies suggest that elective abdominal surgery is recommended for MELD scores < 10 but strongly discouraged for MELD scores > 15 [5]. MELD-based scores are now used to determine liver transplant allocation for patients with end-stage liver disease [5, 6].

CTP and MELD scores, though frequently used, do not consider surgery-specific risks; thus, the Mayo Risk Score (MRS) was developed and rigorously validated as a surgical mortality risk prediction model for patients with cirrhosis [7]. The MRS score was developed between 1980 and 2004 from a single-center cohort of major cardiovascular, orthopedic, and abdominal surgeries. Predictors in this score included the American Society of Anesthesiologists (ASA) physical status classification, international normalized ratio (INR), total bilirubin, creatinine, age, and etiology of liver disease. Despite, MRS being broadly used in clinical practice, there are indications that MRS can overestimate surgical risk, is poorly calibrated, and reflects institution-specific practices that could affect surgical risk [8]. Indeed, the MRS has suffered significant decline in its calibration over time due to improvements in surgical techniques and post-operative care. Although MRS is devised to predict mortality for high-risk cardiovascular, orthopedic, and abdominal surgeries, it does not stratify risk based upon these categories of surgery, each of which imparts unique risks to the patient. In a recent retrospective study, major intra-abdominal surgeries and cardiovascular surgeries had eight- and four-times increased in-hospital mortality, respectively, when compared to orthopedic surgeries. Meanwhile, abdominal wall surgeries and other minimally invasive surgeries tend to be less fatal [9].

The lack of incorporation of the type of surgical procedure into CTP, MELD, and MRS scores may result in an overestimation of risk for minimally invasive or minor surgeries. Moreover, many of these prediction models predate innovations in surgery that have changed the landscape of patient selection and surgical risk, such as the introduction of transcatheter-based valve replacements and other advanced endovascular techniques. These advances have profoundly altered the risk profiles of patients undergoing open procedures. Enhanced perioperative cirrhosis-related medical care and changing demographics of patients with advanced stage of liver disease have also reasonably altered the dynamics of surgical selection and post-operative outcomes.

A parsimonious and improved risk prediction tool is the VOCAL-Penn score that incorporates the type and circumstance of surgery in combination with other readily available clinical data. Predictors included age, pre-operative albumin, platelet count, bilirubin, surgery category, emergency indication, fatty liver disease, American Society of Anesthesiologists (ASA) classification, and obesity (body mass index ≥ 30) [11]. Using a cohort of 3,785 U.S. veterans with cirrhosis who underwent major surgeries, the VOCAL-Penn score has been used to predict 30-, 90-, and 180-day mortality with improved accuracy as compared to traditional methods [11–13]. The VOCAL-Penn had an 87% chance of predicting mortality at 30 days post-surgery, compared to 77% and 72% for the Mayo Risk Score and MELD scores, respectively. This information has enabled the developers of VOCAL-Penn to demonstrate that type of surgical procedure is predictive of post-operative mortality, with open abdominal surgery carrying the highest mortality risk. The VOCAL-Penn score has also proven useful for the stratification of patients at risk of developing infections and further decompensation events post-operatively. When compared with existing clinical standards, the VOCAL-Penn prediction model demonstrated good discrimination for interval decompensation and fair discrimination for post-operative infection, representing significant improvements in prognostication [14].

The potential success of developing future prognostic models for post-operative risk prediction in patients with cirrhosis will heavily rely on feasibility, validity of the proposed models, and ultimately, on front-line healthcare professionals’ usability who can provide the clinical context and workflow. Moreover, the likelihood of developing post-operative complications in cirrhosis is cumulatively linked to pre-existing pre- and peri-operative risk factors. Therefore, future risk prediction algorithms should successfully integrate both pre- and peri-operative factors contributing for both short- and long-term morbidity and mortality. The presence of portal hypertension, for example, should always be considered when assessing risk of abdominal and other technically demanding surgeries. Indeed, hepatic venous pressure gradient (HVPG) has recently been identified as a prognostic factor for cirrhotic patients undergoing surgery. A multicentre study by Reverter et al. demonstrated that HVPG can predict 1-year mortality in patients with cirrhosis undergoing elective extrahepatic surgery (including abdominal, cardiovascular/thoracic, orthopaedic, among others). HVPG values > 16 mmHg, and especially ≥ 20 mmHg, identified a subgroup of patients at very high risk of death (44%) [10]. Reliance on HVPG not only will add a subjective dimension to prediction models but would enable a better risk stratification in surgical patients with cirrhosis and may prove useful for developing prophylactic interventions to improve post-operative outcomes.

The presence of large volume ascites and varices intra-abdominally often deter surgeons from carrying out abdominal surgery. Moreover, the presence of psycho-social risk factors including but not limited to alcohol use and misuse disorder may possibly preclude surgeries [15]. Particular attention, independent of surgical technique, should be given to optimal management of cirrhosis-related complications in the peri-operative period as it is a critical determinant of postsurgical outcomes. Evaluation of frailty as a part of pre-operative workup, for instance, may prove helpful to risk-stratify patients with high risk of post-operative mortality, especially in settings where MELD, MELD-Na, and/or CTP are frequently used for this purpose. A study assessing the impact of baseline frailty for a total of 804 cirrhosis surgeries on post-operative mortality has identified a threshold effect of frailty on post-operative outcomes. Baseline frailty was shown to be an important independent predictor of post-operative mortality, length of stay, and 90-day hospital readmissions [16]. Figure 1 shows the parameters of risk prediction tools currently utilized for assessment of post-operative mortality in patients with cirrhosis.

**Fig. 1:**
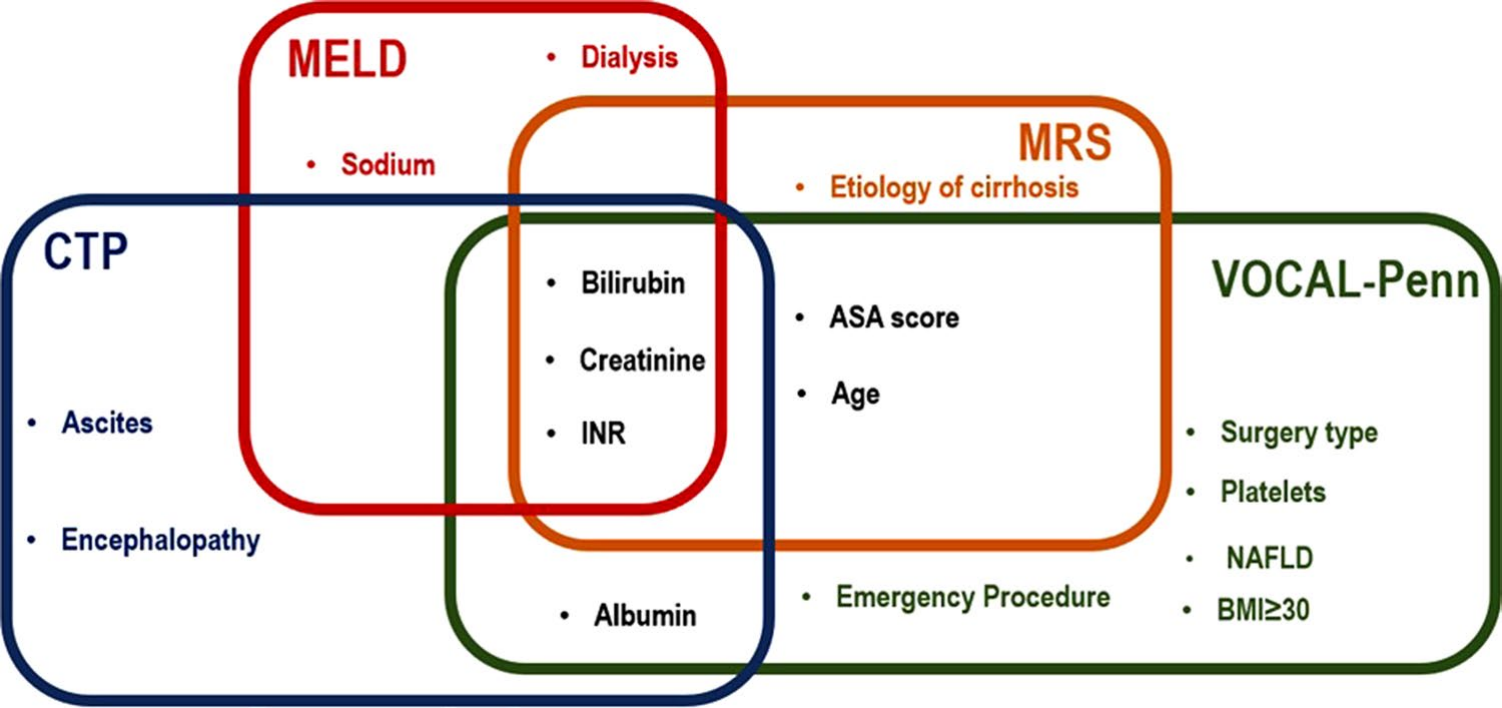
Parameters of risk prediction models utilized for assessment of post-operative mortality in patients with cirrhosis. *ASA* The American Society of Anesthesiologists physical status classification, *BMI* body mass index, *CTP* Child–Turcotte–Pugh, *INR* international normalized ratio, *MELD* Model for End-Stage Liver Disease, *MRS* post-operative Mayo Risk Score, *NAFLD* non-alcoholic fatty liver disease, *VOCAL-Penn* Veterans Outcomes and Costs Associated with the Liver

In summary, existing surgical risk prediction tools for liver cirrhosis are either non-specific, narrow in scope, sub-optimally calibrated or obsolete. Given the absence of a definitive risk stratification system to evaluate operative risk in patients with cirrhosis, it is, therefore, highly recommended to utilize multiple methods [17]. Beyond assessment of existing surgical risk models, it may be valuable to evaluate interventions to mitigate post-operative mortality risk, such as transjugular intrahepatic portosystemic stent shunt insertion, use of non-selective beta blockers, frailty, and nutritional optimization. Ultimately, the decision for surgery should be discussed with the patient and careers in an interdisciplinary clinical environment including the surgeon, anesthetist, and hepatologist. Careful planning, medical and especially nutritional optimization are well known to improve surgical outcomes in patients with advanced liver disease.

Score: Link to online calculator.

CTP: https://www.mdcalc.com/calc/340/child-pugh-score-cirrhosis-mortality

MELD: https://www.mdcalc.com/calc/78/meld-score-model-end-stage-liver-disease-12-older

Original (Pre-2016): https://www.mdcalc.com/calc/2693/meld-score-original-pre-2016-model-end-stage-liver-disease

MRS: https://www.mayoclinic.org/medical-professionals/transplant-medicine/calculators/post-operative-mortality-risk-in-patients-with-cirrhosis/itt-20434721

VOCAL-Penn: https://www.vocalpennscore.com
